# KRAS and NRAS Mutations in Relation to Microsatellite Status in Colorectal Cancer: A Single-Center Study From Romania

**DOI:** 10.7759/cureus.91643

**Published:** 2025-09-05

**Authors:** Ramona Abrudan, Luca Abrudan, Andreea Camarasan, Ovidiu Camarasan, Ovidiu Burta, Ovidiu Pop

**Affiliations:** 1 Department of Psycho-Neurosciences and Rehabilitation, Faculty of Medicine and Pharmacy, University of Oradea, Oradea, ROU; 2 Department of Radiation Oncology, Radiotherapy Laboratory, County Clinical Emergency Hospital Bihor, Oradea, ROU; 3 Department of Morphological Disciplines, University of Oradea, Faculty of Medicine and Pharmacy, Oradea, ROU; 4 Department of Obstetrics and Gynecology, Prof. Dr. Ioan Pușcaș Hospital Șimleu Silvaniei, Șimleu Silvaniei, ROU; 5 Department of Internal Medicine and Pathophysiology, Faculty of Medicine and Pharmacy, University of Oradea, Oradea, ROU; 6 Department of Morphological Disciplines, Faculty of Medicine and Pharmacy, University of Oradea, Oradea, ROU

**Keywords:** colorectal cancer, kras, microsatellite instability (msi), msi/mmr status, mutations, nras

## Abstract

Background/objectives

Colorectal cancer is one of the most prevalent malignancies worldwide, ranking third globally and second in Romania. This study aimed to investigate the coexistence of microsatellite instability (MSI) status with KRAS and NRAS mutations in colorectal cancer patients.

Methods

We analyzed clinicopathological characteristics in 253 patients diagnosed with colorectal cancer between January 1, and June 30, 2024. Polymerase chain reaction (PCR) techniques were used to assess MSI status and detect KRAS and NRAS mutations.

Results

Among the 253 patients, KRAS mutations were detected in 41.1%, while NRAS mutations were identified in 5.1% of cases. Microsatellite stability (MSS) was observed in the majority of cases (95.7%), with only 4.3% of tumors displaying high microsatellite instability (MSI-H). Of all the analyses undertaken to evaluate associations between clinicopathological characteristics of patients with colorectal cancer and KRAS/NRAS or MSS/MSI-H status, tumor localization demonstrated a statistically significant correlation with microsatellite status (p = 0.032).

Conclusions

The prevalence of KRAS and NRAS mutations in the studied population was consistent with international estimates, whereas the frequency of MSI-H tumors was lower compared to other European cohorts. A statistically significant association was observed between tumor localization and MSI status (p = 0.032), with MSI-H tumors confined to colonic sites and MSS tumors predominating in the rectum. These data refine the molecular epidemiological landscape of colorectal cancer in an under-studied population and underscore the necessity of larger multicenter investigations to validate and extend these observations.

## Introduction

Colorectal cancer ranks as one of the most prevalent cancers worldwide, holding the third position globally and second in Romania. According to the World Health Organization, more than 1.9 million new cases were recorded worldwide in 2020, with Romania reporting 12,968 new cases [1]. Globally, colorectal cancer is the second leading cause of cancer-related mortality. Therefore, a more accurate analysis of its pathogenesis, particularly the molecular profile of this disease, is essential [1-5].

The incidence of colorectal cancer is higher in men than in women and occurs more frequently after the age of 50. However, elderly individuals have a significantly higher predisposition to developing this disease [4,6-9].

In 1990, Bufill first described two distinct categories of colorectal tumors: right-sided and left-sided tumors, each with different molecular and clinical characteristics. The most plausible explanation for this difference lies in the distinct embryological origins of the colon, with the right colon deriving from the midgut and the left colon from the hindgut, and also in the blood supply [10,11]. Based on this classification, the right colon includes the cecum, ascending colon, and the first two-thirds of the transverse colon, whereas the left colon consists of the distal third of the transverse colon, descending colon, and sigmoid colon [10-12].

Colorectal cancer is an oncological pathology that arises from various alterations, which may be the consequence of external factors (such as diet, tobacco use, and alcohol consumption) as well as internal factors, either hereditary or resulting from changes in intracellular signaling pathways caused by genetic and epigenetic alterations [6-13].

Women are more likely to develop tumors in the right colon, while men more frequently present with tumors in the left colon [10,14,15]. Clinically, right-sided colon tumors tend to be larger and are often associated with anemia and rapid weight loss. In contrast, left-sided colon tumors are typically more infiltrative, commonly leading to rectal bleeding and bowel obstruction [10,14-16]. Building on Bufill’s study, recent research has further demonstrated that right-sided colon tumors exhibit higher activation of the MAPK and mTOR pathways, microsatellite instability (MSI), and the CpG island methylator phenotype. In contrast, left-sided colon tumors show activation of the EGFR/Wnt pathway and chromosomal instability [10,14,15].

The most significant signaling pathways involved in the development of colorectal cancer are the RAS/RAF/MAPK pathway and the PI3K/PTEN/AKT/mTOR pathway, both of which are activated by a ligand binding to the epidermal growth factor receptor (EGFR) [6,17-20]. Their activation leads to uncontrolled cell proliferation, increased cellular motility, and resistance to programmed cell death. Key proto-oncogenes associated with these pathways include members of the RAS family (KRAS, NRAS, HRAS) and PIK3CA [6,17-20].

In colorectal cancer, the most frequent mutations are observed in the KRAS gene (Kirsten rat sarcoma viral oncogene homolog), found in approximately 45% of cases, followed by the NRAS gene (neuroblastoma rat sarcoma viral oncogene homolog), found in about 5% of cases, and the PIK3CA gene, found in about 10% of cases [6,17-20]. KRAS mutation status is an important prognostic marker, with current therapies providing a median survival of about two years in mutated patients compared to those with wild-type tumors [21].

Mutations in the proto-oncogenes KRAS and NRAS are commonly located in exon 2 (codons 12 and 13), exon 3 (codons 59 and 61), and exon 4 (codons 117 and 146) [6,17].

Microsatellites are short tandemly repeated DNA sequences dispersed throughout the genome, which are particularly prone to replication errors. The DNA mismatch repair (MMR) system is responsible for correcting these errors and maintaining genomic stability. MSI arises when the MMR system is deficient, leading to length alterations in microsatellite sequences rather than direct mutations in the MMR genes themselves [18,22-25]. Depending on the number of affected genes, MSI is classified as follows: MSI-H (microsatellite instability-high), when two or more genes are mutated; MSI-L (microsatellite instability-low), when only one gene is affected; and MSS (microsatellite stable), when no genes are affected [18,22-25].

MSI is identified in approximately 15% of colorectal cancer cases. The prevalence of MSI varies according to the disease stage: in stage II colorectal cancer, around 20% of patients demonstrate MSI-H, whereas in stage III, this proportion decreases to approximately 12%. The lowest prevalence is observed in stage IV, where it is around 4% [22,25-27].

Aim and scope

The principal aim of this study is to estimate the prevalence of KRAS and NRAS mutations among colorectal cancer cases according to microsatellite status (MSI-H versus MSS) in a Romanian cohort. Additionally, the study explores the associations of the mutations with tumor grade, tumor location, and patient age, and describes the distribution of MSI-H and MSS status in the studied population.

## Materials and methods

Case selection

**Figure 1:**
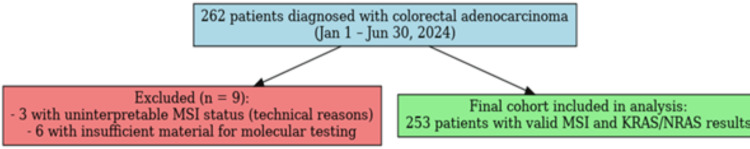
Patient inclusion and exclusion criteria for the selected cohort

The presented study was designed as a retrospective, observational analysis. Between January 1 and June 30, 2024, all patients with a histopathologically confirmed diagnosis of colorectal cancer who presented to Resident Laboratory, Bihor County, Romania, a laboratory with recognized expertise in this domain, were considered for inclusion. The Resident Laboratory serves a broad geographical area of Romania and conducts analyses for KRAS/NRAS mutations and MSI status. We initially identified 262 cases with a confirmed diagnosis of colorectal cancer. After applying the exclusion criteria, 253 cases were eligible for inclusion in the final cohort (Figure 1).

Inclusion Criteria

This study included all patients diagnosed with colorectal adenocarcinoma between January 1 and June 30, 2024, with histopathological confirmation of the disease, regardless of sex or age. Eligible cases were those whose tumor samples were processed at Resident Laboratory, which applies standardized protocols for KRAS and NRAS mutation testing, as well as MSI status. Only cases with formalin-fixed paraffin-embedded (FFPE) tumor tissue of adequate quality and quantity for molecular analysis were included.

Exclusion Criteria

Patients were excluded if they lacked histopathological confirmation of colorectal adenocarcinoma or if their tumor samples were unsuitable for molecular testing (e.g., degraded FFPE tissue, insufficient tumor cell content <20%). Additional exclusions applied to cases with missing molecular data (no results for KRAS, NRAS, or MSI status), duplicate/recurrent specimens from the same patient, or documented withdrawal/lack of consent.

All tissue samples were obtained from hospitals where patients had undergone diagnostic investigations and received a confirmed diagnosis of colorectal cancer. Informed written consent was obtained from all patients. All the specimens were transported under proper conditions and processed at the Resident Laboratory.

Ethical Considerations

This study was conducted in full accordance with the ethical principles outlined in the Declaration of Helsinki and was approved by the Local Ethics Commission for Clinical and Research Developmental Studies, Resident Laboratory, Oradea, Romania (approval number 11, issued on December 20, 2024).

Method

Histopathology, DNA Extraction, and Quality Control

Tumor cellularity was assessed on H&E-stained slides, with a minimum threshold of ≥20% tumor cells, and macrodissection was applied in selected cases to increase tumor content [28]. DNA concentration and integrity were verified by automated fluorescent electrophoresis on an Agilent 2200 TapeStation (Agilent Technologies, Santa Clara, CA, USA), a validated platform for FFPE-derived DNA quality control. Analyses were performed under internal SOPs aligned with manufacturer’s protocols; duplicate testing and external positive/negative controls were not applied, as the assay includes internal quality control reactions and reproducibility has been independently validated [28,29].

KRAS and NRAS Genotyping

KRAS and NRAS genotyping was performed using a real-time polymerase chain reaction (PCR) assay, the AmoyDx® KRAS/NRAS Mutations Detection Kit (Amoy Diagnostics, Xiamen, China; Cat. No. ADx-KRAS/NRAS-32), which detects 32 hotspot mutations across codons 12, 13, 59, 61, 117, and 146. DNA was extracted from FFPE tumor samples using the QIAamp DNA FFPE Tissue Kit (Qiagen, Hilden, Germany; Cat. No. 56404), and amplification was carried out on an ABI 7500 Fast Real-Time PCR System (Applied Biosystems) according to the manufacturer’s protocol. Mutation calls were automatically generated by the AmoyDx proprietary software using predefined Ct thresholds [30].

Microsatellite Instability Testing

MSI status was determined using a five-marker mononucleotide PCR panel (BAT-25, BAT-26, NR-21, NR-24, MONO-27; Promega MSI Analysis System v1.2) [31]. DNA was extracted from FFPE tumor tissue using the QIAamp DNA FFPE Tissue Kit (Qiagen, Cat. No. 56404), according to the manufacturer’s protocol. PCR reactions were prepared in a 25-μL volume with standard reagent concentrations. PCR amplification was performed with an initial denaturation at 95°C for 10 min, followed by 35 cycles of denaturation at 95°C for 30 s, annealing at 55°C for 30 s, and extension at 72°C for 30 s, with a final extension at 72°C for 10 min. Amplicons were analyzed by capillary electrophoresis on an ABI Prism 3130xl Genetic Analyzer, and fragment analysis was performed using GeneMapper software v5.0. Tumors were classified as MSI-H if instability was present in ≥2 loci, MSI-L if present in one locus, and MSS if no instability was observed, according to established guidelines [31,32].

Statistical analysis

All data were entered into Microsoft Office Excel 2016 (Microsoft Corp., Redmond, WA), and the results were analyzed using the Statistical Package for the Social Sciences Version 26 (IBM Corp., Armonk, NY). The data were organized into ordinal or nominal variables. The obtained results were evaluated using chi-square tests, Pearson coefficients, and Eta coefficients. A p-value of less than 0.05 was considered statistically significant, with a 95% confidence interval.

## Results

Clinicopathological aspects of patients with colorectal cancer

We analyzed a cohort of 253 patients, primarily from the western, northwestern, and central regions of Romania, with sporadic cases from other areas of the country. The prevalence of colorectal cancer was higher among males, accounting for 67.2% cases, compared to females, who represented only 32.8% (Table 1). Regarding the age of onset, the majority of cases were observed in the age group of 61-70 years, 96 (37.9%) patients, closely followed by the age group of 71-80 years, which comprised 80 (31.6%) patients (Table 1). In terms of tumor localization, rectal tumors were the most common, 95 (37.5%) patients, followed by the left colon (splenic flexure, descending colon, sigmoid colon), 79 (31.2%) patients, and right colon (cecum, ascending colon, hepatic flexure, transverse colon), 70 (27.6%) patients (Table 1). Histologically, all cases were adenocarcinomas. From a tumor grading perspective, the majority were moderately differentiated (G2), comprising 201 (79.4%) of the cases studied. Another important parameter assessed was the stage of the disease (Table 1). Unfortunately, the majority of cases were stage IV, representing 158 (62.5%) of the total cases examined.

**Table 1:** Clinicopathological aspects of the patients with CRC. Colorectal cancer demonstrated a higher prevalence in males, predominantly affected individuals aged 61–70 years, with rectal tumors being most common and the majority of cases diagnosed at stage IV. CRC, colorectal cancer

Clinicopathological aspects of patients with CRC	Number (%)
Gender	
Male	170 (67.2%)
Female	83 (32.8%)
Age by decade	
30-40	5 (2%)
41-50	20 (7.9%)
51-60	41 (16.2%)
61-70	96 (37.9%)
71-80	80 (31.6%)
81-90	11 (4.3%)
Localization	
Right colon	70 (27.6%)
Left colon	79 (31.2%)
Rectum	95 (37.5%)
Metastasis (with occult primary tumor)	6 (2.4%)
Double localization	2 (0.8%)
Vermiform appendix	1 (0.4%)
Tumor grading	
G1	18 (6.3%)
G2	201 (79.4%)
G3	31 (12.3%)
G4	3 (1.2%)
Staging	
1	0
II	16 (6.3%)
III	79 (31.2%)
IV	158 (62.5%)

KRAS/NRAS mutations and MMR status in the studied patient cohort

**Figure 2:**
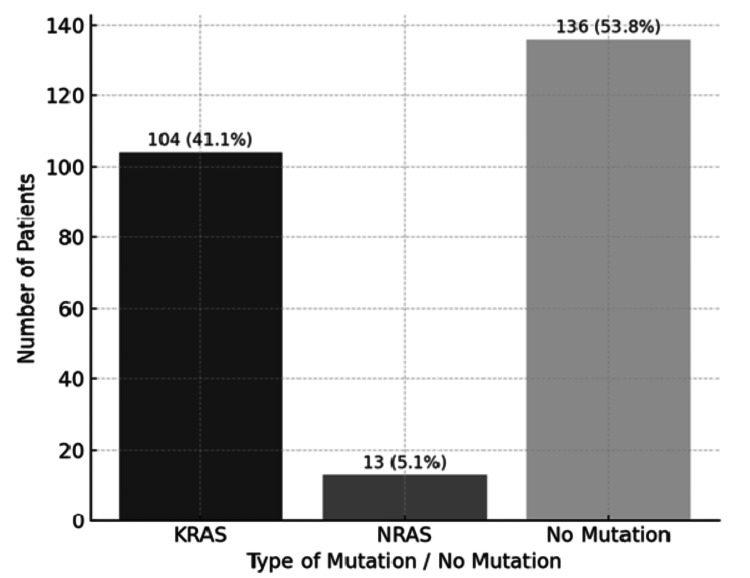
RAS status among colorectal cancer patients in the selected cohort: 53.8% of the patients included in the study had no detectable mutation (wild type), whereas 41.1% harbored a KRAS mutation.

Among the 253 patients studied, 104 (41.1%) cases presented KRAS mutations, in contrast to 13 (5.1%) cases with NRAS mutations, and the remaining patients in the cohort were wild type (no mutation detected), 136 (53.8%) (Figure 2).The activating mutation in the KRAS gene was c.35G>A (Gly12Asp), observed in 29.1% of cases, and exon 2 was the most affected, observed in 75.2% of cases (Tables 2, 3). On the other hand, in the NRAS gene, the c.181C>A (Gln61Lys) mutation was observed in 3.4% of patients, and exon 3 was the most identified, observed in 6.8% of cases (Tables 2, 3). Regarding gender distribution among patients with KRAS mutations, males were more prevalent (28.5%) compared to females (12.6%). In contrast, colorectal cancer patients with NRAS mutations displayed a nearly equal gender distribution, with 2.4% male and 2.8% female. In terms of age, KRAS mutations were most frequently observed in the age group of 71-80 years, with 35 (13.8%) patients, while NRAS mutations were most common in the age group of 61-70 years, with eight (3.2%) patients. Regarding tumor localization, rectal cancer was more prevalent and was associated with KRAS mutations in 43 (17%) cases and NRAS mutations in 8 (3.2%) patients. Stage IV disease was the most prevalent, observed in 64 (25.3%) cases with KRAS mutations and 10 (4%) cases with NRAS mutations. From a tumor grading perspective, moderately differentiated (G2) tumors were the most common, accounting for 32.8% of cases in patients with KRAS mutations and 11 (4.3%) cases in patients with NRAS mutations.

**Table 2:** Activating mutations. The most common KRAS activating mutation was c.35G>A (Gly12Asp), observed in 29.1% of cases. Chi-square test, p=0.0001

Mutation		KRAS	NRAS	Total
c.34 G>T Gly12Cys	Count	5	0	5
	% of total	4.3%	0.0%	4.3%
c.34gG>C Gly12Arg	Count	2	0	2
	% of total	1.7%	0.0%	1.7%
c.34G>A Gly12Ser	Count	4	0	4
	% of total	3.4%	0.0%	3.4%
c.35G>C Gly12Ala	Count	2	2	4
	% of total	1.7%	1.7%	3.4%
c.35G>TGly12Val	Count	17	1	18
	% of total	14.5%	0.9%	15.4%
C.37G>TGly13Cys	Count	2	0	2
	% of total	1.7%	0.0%	1.7%
c.35G>A Gly12Asp	Count	34	3	37
	% of total	29.1%	2.6%	31.6%
c.38G>A Gly13Asp	Count	21	1	22
	% of total	17.9%	0.9%	18.8%
C.34_35GG>TTGLY12PHE	Count	1	0	1
	% of total	0.9%	0.0%	0.9%
c.175G>A Ala59Thr	Count	1	0	1
	% of total	0.9%	0.0%	0.9%
c.182A>T Gln61Leu	Count	1	1	2
	% of total	0.9%	0.9%	1.7%
c.183A>C Gln61His	Count	2	1	3
	% of total	1.7%	0.9%	2.6%
c.181C>A Gln61Lys	Count	0	4	4
	% of total	0.0%	3.4%	3.4%
c.182A>Gln61Arg	Count	0	2	2
	% of total	0.0%	1.7%	1.7%
c.436G>A Ala146Thr	Count	9	0	9
	% of total	7.7%	0.0%	7.7%
c.436gG>A Ala146Pro	Count	1	0	1
	% of total	0.9%	0.0%	0.9%
Total	Count	102	15	117
	% of total	87.2%	12.8%	100.0%

**Table 3:** Activating exons. The highest frequency of mutations was observed in exon 2. p=0.0001

Exon	KRAS	NRAS
Exon 2	88 (75.2%)	7 (6.0%)
Exon 3	4 (3.5%)	8 (6.8%)
Exon 4	10 (8.5%)	0 (0%)

**Figure 3:**
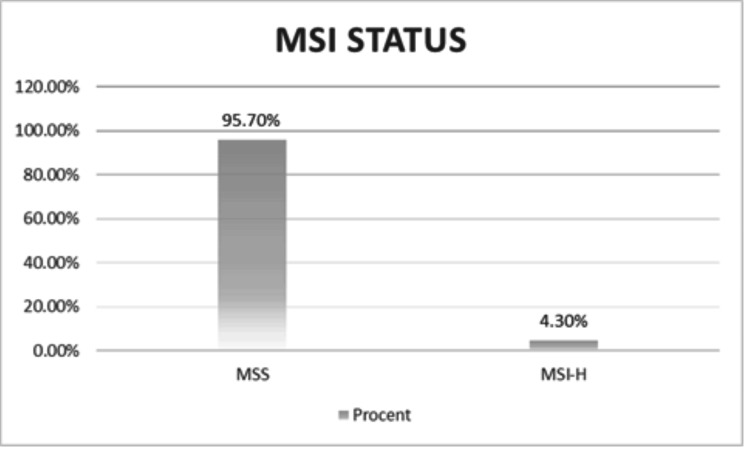
MSI status. MSS was present in 95.7% of cases, while MSI-H was seen in 4.3%. MSI, microsatellite instability; MSI-H, high microsatellite instability; MSS, microsatellite stability

Regarding MSI status, MSS was observed in 242 (95.7%) cases, and only 11 (4.3%) patients exhibited MSI-H (Figure 3). Among patients with MSS status, male patients accounted for 165 (65.2%) cases compared to 77 (30.4%) female patients. In terms of age distribution, the age group of 61-70 years demonstrated the highest representation, 92 (36.4%) cases. Regarding tumor localization, rectal tumors were most frequently associated with stable MSI status, identified in 95 (37.5%) cases (Table 4). The disease stage at the time of MSS assessment was predominantly stage IV, observed in 151 (59.7%) cases. From a histopathological grading perspective, moderately differentiated tumors were the most commonly observed, accounting for 193 (76.3%) cases. Among MSI-H cases, males constituted 2.0% and females constituted 2.4%. Regarding age distribution, the age groups of 51-60 and 61-70 years each comprised four patients (1.6% per group). In terms of tumor localization, the most frequent sites were the right and left colon, with three cases each (1.2% per site) (Table 4). Most cases were diagnosed at an advanced stage (stage IV, 2.8%), and the majority of tumors were moderately differentiated (G2, 3.2%).

**Table 4:** Correlation between localization of tumor and MSI status demonstrating statistical significance (p=0.032), suggesting a potential association between rectal tumors and MSS status. p=0.032 MSI, microsatellite instability; MSI-H, high microsatellite instability; MSS, microsatellite stability

Localization	Number/percentage (n/%)	MSS	MSI-H	Total
Cecum	Count	14	2	16
	% of total	5.5%	0.8%	6.3%
Ascending colon	Count	40	3	43
	% of total	15.8%	1.2%	17.0%
Transverse colon	Count	9	2	11
	% of total	3.6%	0.8%	4.3%
Descending colon	Count	33	3	36
	% of total	13.0%	1.2%	14.2%
Sigmoid colon	Count	43	0	43
	% of total	17.0%	0.0%	17.0%
Rectum	Count	95	0	95
	% of total	37.5%	0.0%	37.5%
Metastasis	Count	5	1	6
	% of total	2.0%	0.4%	2.4%
Cecum and sigmoid colon	Count	1	0	1
	% of total	0.4%	0.0%	0.4%
Jejunum and sigmoid colon	Count	1	0	1
	% of total	0.4%	0.0%	0.4%
Vermiform appendix	Count	1	0	1
	% of total	0.4%	0.0%	0.4%
Total	Count	242	11	253
	% of total	95.7%	4.3%	100.0%

We investigated potential associations between MSI status and several clinicopathological variables, including sex, age, disease stage, and histologic grade, using chi-square tests. None of these comparisons reached statistical significance (all p > 0.05) (Table 5). In contrast, the distribution of tumor location by MSI status showed a significant association: rectal cancers were more frequent among MSS cases, achieving statistical significance (p = 0.032) (Table 4).

**Table 5:** Association between KRAS/NRAS mutations and MSI status. Chi-square test, p=0.38 MSI, microsatellite instability; MSI-H, high microsatellite instability; MSS, microsatellite stability

MSI status	Number/percentage (n/%)		KRAS	NRAS	No mutation	Total
	MSS	Count	101	13	128	242
		% of total	39.9%	5.1%	50.6%	95.7%
	MSI-H	Count	3	0	8	11
		% of total	1.2%	0.0%	3.2%	4.3%
Total		Count	104	13	136	253
		% of total	41.1%	5.1%	53.8%	100.0%

## Discussion

Colorectal cancer is a heterogeneous oncological pathology, frequently encountered both within our country and worldwide. The diverse mechanisms underlying the disease's development, particularly genetic alterations, have led to the identification of biomarkers with diagnostic and prognostic significance, including KRAS/NRAS mutations and MSI status [18,32-34].

In the broader cohort of 253 patients analyzed with the histopathological result of adenocarcinoma, 41.1% patients presented KRAS mutations, while 5.1% patients presented with NRAS mutations. In this context, comparing our general findings with data from the specialized literature reveals a concordance regarding the proportion of KRAS and NRAS mutations. KRAS mutations are reported in 35-45% of colorectal cancer cases, while NRAS mutations occur in 1-6% of cases [6,17,19,32,34,35]. Of the 253 patients, 242 (95.7%) patients exhibited an MSS status and 11 (4.3%) exhibited an MSI-H status. This prevalence is notably lower compared to data reported in the literature, which indicates higher frequencies among Europeans (5-24%), Caucasian Americans of European descent (8-20%), African Americans (12-45%), and Egyptians (37%). Conversely, populations from Asian countries, particularly China and Japan, exhibit markedly reduced incidence rates, ranging from 3.8% to 10% [18,34,36,37]. Upon assessing the proportion of patients with MSS within our study cohort, a notably lower incidence was observed compared to other European countries, yet it remains relatively comparable to the rates reported in Asian populations.

To our best knowledge, this investigation is among the first in Romania to assess the possible relation between KRAS or NRAS mutations in combination with MSI status in patients with colorectal cancer.

Regarding the activating mutation in the KRAS gene, the most frequently observed variant was c.35G>A (Gly12Asp), identified in 29.4%, followed by c.38G>A (Gly13Asp), detected in 17.6%. Both mutations were located within exon 2. Regarding this mutation, our findings align with those reported in the literature, with both European and Asian studies confirming the same mutation localization [6,34,36,38]. This consistency suggests that the global distribution of populations does not significantly influence this characteristic.

In terms of gender distribution, a clear predominance of colorectal cancers with KRAS mutations was observed in males, accounting for 28.5% compared to 12.6% of females. These findings align with the well-established observation that the incidence of colorectal cancer is higher among males [1,5,8].

From an age perspective, while it is well-established that colorectal cancer is more prevalent after the age of 50, as previously mentioned, the majority of studies conducted in European, Asia, or even African cohorts reported a median age of onset around 65 years [18,34,36,39-41]. In our cohort, age-stratified analysis revealed heterogeneity in RAS mutational status. The greatest frequency of KRAS mutations occurred among patients aged 71-80 years (n = 35; 13.8% of the cohort).

When analyzing tumor localization, our findings reveal a higher prevalence of rectal cancers associated with KRAS mutations, observed in 17% of cases, compared to left-sided colon cancers, which accounted 10.6%, and right-sided colon cancers, representing 12.3% of cases. The reported data in existing studies show variability; for example, Kawazoe et al. reported a notable association between KRAS mutations and rectal cancers, and similarly, El Agy et al. documented KRAS mutations in left-sided colon cancers, while Zhang et al. highlighted their presence in right-sided colon cancers [42-44]. These findings reflect the heterogeneous nature of KRAS-associated colorectal cancer localization patterns.

When analyzing disease staging, the majority of cases were unfortunately identified at stage IV, accounting 62.5%. Previous studies conducted over time have not demonstrated a statistically significant correlation, and only one study did highlight that older patients are more frequently diagnosed at stage IV [18].

From the perspective of tumor grading, moderately differentiated colorectal cancers (G2) are the most frequently observed, representing a proportion of 79.4% of the total cases. While many studies have not shown a statistically significant correlation, Bisht et al. did find one [33].

Mutations in the NRAS gene are extremely rare, and limited data are available in the scientific literature [39,43-46]. In our study, the most frequent activating mutation identified was c.181C>A Gln61Lys in exon 3, observed in 3.4% of patients. In agreement with the findings of Jauhri et al., the prevalence of this mutation was slightly higher among females in our cohort (2.8%) than males (2.4%) [47].

In terms of similarities with the findings of Jauhri et al., the first resemblance lies in tumor location, which predominantly involves distal sites. The NRAS mutation was observed in 3.2% of rectal cancer. The second similarity pertains to the location of the activating mutation in the NRAS gene, specifically at codon 61 [47].

By contrast, NRAS-mutant cases were most frequent in the age group of 61-70 years (n = 8; 3.2%).

In terms of age distribution, patients with MSS status were predominantly in the age group of 61-70 years, accounting for 36.4%, followed by those in the age group of 71-80 years, accounting for 30.4%. When stratified by age, MSI-H status was represented by four cases in the age group of 51-60 years, four cases in the age group of 61-70 years, and three cases in the age group of 71-80 years. According to the study by Wang et al., a higher incidence of MSI-H status was observed in younger patients compared to those with MSS. However, in the study by Niu et al., MSI-H status was more prevalent in older patients, although this analysis was limited to individuals with stage III colorectal cancer [24].

In the MSS subset, the sex distribution favored males (65.2%) over females (30.4%). By contrast, MSI-H tumors were observed in 2.4% of females and 2.0% of males. This pattern is broadly concordant with prior reports suggesting a female predominance of MSI-H disease; however, the differences were modest and did not achieve statistical significance in our cohort, nor has this association been consistently replicated across studies [18,23,47].

Regarding the localization of colorectal tumors with MSS status, a significant proportion were identified as rectal cancers, constituting a proportion of 37.5%, followed by left-sided colon cancers, accounting for 30%, and right-sided colon cancers, accounting for 24.9%. In contrast, in patients with MSI-H status, right-sided colon cancer, left-sided colon cancer, and occult tumors were each observed in equal proportions, each comprising 2% of the cases, with no cases of rectal cancer reported. Statistical analysis yielded a significant association (p = 0.032), suggesting a potential predisposition for rectal cancers in the context of MSS status. Although the sample size is limited, it may be cautiously extrapolated that MSI-H status is more frequently associated with colon tumors. Available evidence indicates that MSI-H status is predominantly associated with right-sided colon cancers, whereas MSS is more prevalent in left-sided colon and rectal cancers, as also observed in the study by Niu et al [24]. Furthermore, Kassem et al. identified three cases of rectal cancer and MSI-H status in their investigation of the Egyptian population, although these findings lacked statistical significance [19].

From the perspective of disease staging, the majority of cases were identified in stage IV (59.7%), followed by stage III (30%) and stage II (5.9%), in the MSS group. On the other hand, the majority of cases with MSI-H status were stage IV (2.8%). In comparison with data from the literature, where MSI-H status has been shown to correlate with advanced stages, our findings align with these trends [18,47,48]. Nevertheless, findings from Formica et al. underscore a heightened tumor aggressiveness linked to the coexistence of these mutations, spanning from early-stage disease to advanced metastatic progression [49].

In light of the high KRAS mutation frequency and substantial MSS representation, we evaluated their association, but the analysis did not reach statistical significance. In the meta-analysis by Ashktorab et al., a higher incidence of colorectal cancers with MSS status and KRAS mutation was observed in the African-American population compared to the Caucasian population [50]. Some investigations have demonstrated that the combination of KRAS mutations and MSS status is associated with increased tumor aggressiveness, a poorer prognosis, and lower overall survival rates compared to MSS tumors without KRAS mutations [35,49-52]. Li et al. discovered that KRAS activation was shown to be an important mediator of tumor cell invasion and metastasis in MSS tumors [53].

Limitations of the study

This study has several limitations that are further presented. First, the study is a single-center design, and this aspect may limit the generalizability of the findings to the broader Romanian population. Although the Resident Laboratory serves a wide geographical area, the results could still be influenced by specific referral patterns, technical protocols, and patient demographics associated with our center. Second, the retrospective design relies on existing medical records and laboratory data, which may be incomplete or lack certain clinicopathological variables. Third, while the revised analysis is based on the entire cohort of 253 patients, the number of cases with certain rare molecular profiles, such as the coexistence of MSI-H and KRAS/NRAS mutations, remains small, limiting the statistical power for subgroup comparisons. Fourth, because the study was conducted in a private pathology laboratory, we were unable to follow patients longitudinally or collect survival rate data, which limits the ability to correlate molecular findings with long-term outcomes.

## Conclusions

The study provides the first comprehensive evaluation of KRAS and NRAS mutational status in conjunction with MSI in a Romanian colorectal cancer cohort. Significant association was observed between tumor localization and MSI status, with MSI-H tumors localized to colonic sites and MSS tumors predominating in the rectum. No significant link was identified between RAS mutations and MSI status, a result likely reflecting the low number of MSI-H cases. Larger, multicenter studies in Romania and beyond are warranted to further elucidate potential molecular profiles in colorectal cancer, in conjunction with several public awareness campaigns on colorectal cancer, aimed at reducing the high incidence of patients diagnosed at stage IV disease.
